# Effects of Passive Finger Movement on Cortical Excitability

**DOI:** 10.3389/fnhum.2017.00216

**Published:** 2017-05-03

**Authors:** Masaki Nakagawa, Ryoki Sasaki, Shota Tsuiki, Shota Miyaguchi, Sho Kojima, Kei Saito, Yasuto Inukai, Hideaki Onishi

**Affiliations:** Institute for Human Movement and Medical Sciences, Niigata University of Health and WelfareNiigata, Japan

**Keywords:** passive movement, transcranial magnetic stimulation, motor evoked potential, primary motor cortex, afferent facilitation

## Abstract

This study examined the effects of joint angle and passive movement direction on corticospinal excitability. The subjects were 14 healthy adults from whom consent could be obtained. We performed two experiments. In Experiment 1, we measured motor evoked potential (MEP) amplitude, F-wave and M-wave at 0° and 20° adduction during adduction or abduction movement, in the range of movement from 10° abduction to 30° adduction. In Experiment 2, MEPs were measured at static 0° and 20° adduction during passive adduction from 10° adduction to 30° adduction and static 20° adduction. MEP, F-waves and M-waves were recorded from the right first dorsal interosseous (FDI) muscle. Experiment 1 revealed significantly increased MEP amplitude at 0° during passive adduction compared to static 0° (*p* < 0.01). No other significant differences in MEP, M-wave and F-wave parameters were observed. In Experiment 2, MEP amplitude was significantly higher at 20° adduction during passive adduction compared with static 0° (*p* < 0.01). Based on these findings, it appears that fluctuations in MEP amplitude values during passive movement are not influenced by joint angle, but rather it is possible that it is due to intracortical afferent facilitation (AF) dependent on afferent input due to the start of movement and interstimulus interval (ISI) of transcranial magnetic stimulation (TMS).

## Introduction

Passive movement is known to alter the excitability of sensorimotor cortex. Many studies have reported that the primary somatosensory cortex (S1) and primary motor cortex (M1) are also activated during afferent input from the periphery associated with passive movement (Xiang et al., 1997; Carel et al., 2000; Reddy et al., 2001; Onishi et al., 2013; Shriver et al., 2013). Moreover, it has been reported that motor evoked potentials (MEPs) evoked by transcranial magnetic stimulation (TMS) significantly decrease during passive movement in the direction of muscle extension (Lewis et al., 2001; Edwards et al., 2002, 2004; Coxon et al., 2005; Chye et al., 2010) and significantly increase with passive movement in the direction of muscle shortening (Lewis et al., 2001; Coxon et al., 2005; Chye et al., 2010). Moreover, Lewis et al. (2001) reported MEP changes in response to normalized static MEP values corresponding to the joint angles in the change of motion direction.

An examination of the relationship between joint angle and MEP amplitude during passive movement of the wrist revealed that MEPs decrease immediately after the start of extension (Lewis et al., 2001). In contrast, it was reported that MEPs do not decrease immediately after passive index finger adduction from the abduction position but do decrease at 0° (mid-position; Edwards et al., 2002, 2004). Thus, the relationship between joint angle and MEP amplitude during passive movement is inconsistent across studies. Further, there are no reports concerning the relationship of joint angle and MEP during passive movement in the direction of both muscle extension and shortening.

In contrast, multiple studies have reported consistent MEP changes in response to TMS of the M1 at specific intervals following peripheral nerve electrical stimulation. For example, when the interstimulus interval (ISI) between median nerve electrical stimulation and TMS was set at 20–40 ms, MEP amplitude decreased—a response termed short latency afferent inhibition (SAI; Tokimura et al., 2000; Kotb et al., 2005; Tamburin et al., 2005; Devanne et al., 2009). Alternatively, at 45–80 ms ISI, afferent facilitation (AF) of MEP amplitude was observed (Deletis et al., 1992; Komori et al., 1992; Devanne et al., 2009; Degardin et al., 2011). These changes are attributed, respectively, to decreased (SAI) and increased (AF) excitability of the corticospinal pathway. In contrast to electrical peripheral stimulation paired with TMS, there are no studies on SAI and AF during passive movement (i.e., in response to movement-associated afferent activation).

It is possible that passive movement could alter corticospinal tract excitability by activating afferent inputs dependent on direction of movement, specific joint angle, or both, and that these changes would be manifested as SAI or AF according to ISI. Therefore, the principal aim of the present study was to elucidate the effects of joint angle and passive movement direction on corticospinal tract excitability by measuring TMS-evoked MEPs.

## Materials and Methods

### Subject

Fourteen healthy subjects (10 males and 4 females; mean ± standard deviation, 23.2 ± 3.5 years; age range, 20–32 years) participated in both the experiments in this study. None of the participants had a history of neuromuscular or orthopedic disease, and all of them gave written informed consent to participate. The study conformed to the Declaration of Helsinki and International Code of Medical Ethics of the World Medical Association and was approved by the ethics committee at the Niigata University of Health and Welfare, Niigata, Japan.

### Experimental Procedures

This study comprised Experiment 1 and Experiment 2. During both experiments, the subjects were seated comfortably in a reclining chair. Slight elbow flexion was performed at rest with the right forearm placed in a pronated position on a stand with the passive movement device attached to the right index finger.

### Experiment 1–Measurement of MEPs, M-waves and F-waves of during Passive Movement

**Figure 1:**
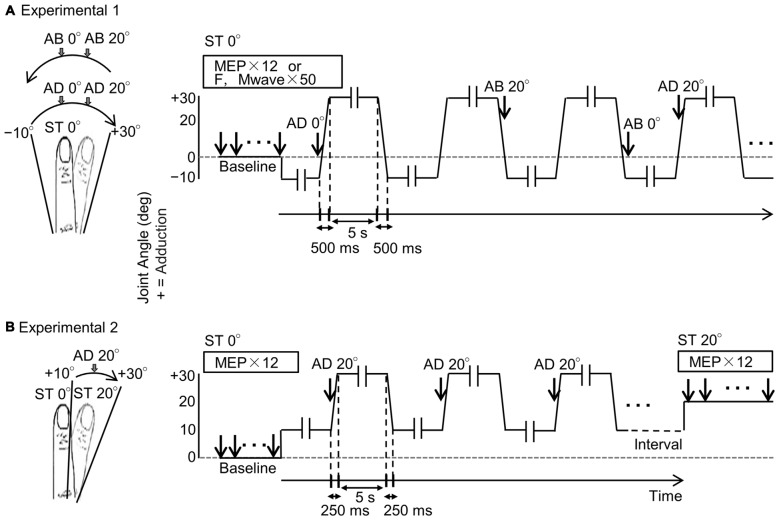
**Experimental protocols. (A)** Schema of Experiment 1. The left diagram shows the right index finger and its range of movement, and the right diagram shows joint angle and measurement timings for motor evoked potentials (MEPs), F-waves and M-waves during passive movement. The vertical and horizontal axes represent joint angle and time, respectively. MEPs, M-waves and F-waves were measured randomly in one adduction–abduction cycle in only one condition. Velocity of the passive movement was 80°/s and the range of movement was from 10° abduction to 30° adduction. Movement from 10° abduction to 30° adduction took 500 ms and a 5-s pause was included between changes in direction (-ll-). MEPs, M-waves and F-waves were measured on different days. Baseline (ST 0°): 0° static joint angle; AD 0°: 0° joint angle during passive adduction; AD 20°: 20° adduction (joint angle) during passive adduction; AB 20°: 20° adduction (joint angle) during passive abduction; AB 0°: 0° joint angle during passive abduction. **(B)** Schema of Experiment 2. The diagrams correspond to those in **(A)**. The MEP was measured only once during a single adduction–abduction cycle at 20° adduction during passive adduction from 10° to 30°. Baseline (ST 0°): 0° static joint angle; AD 0°: 0° joint angle during passive adduction; ST 20°: 20° static adduction angle. Velocity of passive movement was 80°/s. Movement from 10° abduction to 30° adduction took 250 ms (125 ms from 10° adduction to 20° adduction) with 5-s pauses (-ll-) between changes in direction.

MEPs were measured 12 times from the right first dorsal interosseous (FDI) muscle in response to TMS at static 0° joint angle. Then, MEPs were measured 12 times while the right index finger was subjected to passive adduction movement from 10° abduction to 30° adduction and passive abduction from 30° adduction to 10° abduction (Figure 1A). This same protocol was repeated on separate days to measure 50 individual M-waves and F-waves in response to ulnar nerve stimulation.

### Experiment 2–MEP Measurement at Static Joint Angles and during Passive Movement

After measuring 12 MEPs at static 0°, the range of passive movement was set from 10° to 30° adduction and MEPs measured 12 times at 20° adduction during movement. Subsequently, MEPs were measured 12 times at static 20° adduction (Figure 1B). Measurements of MEPs at 20° adduction under movement and static conditions were conducted at intervals of 10 min.

### Passive Movement

**Figure 2:**
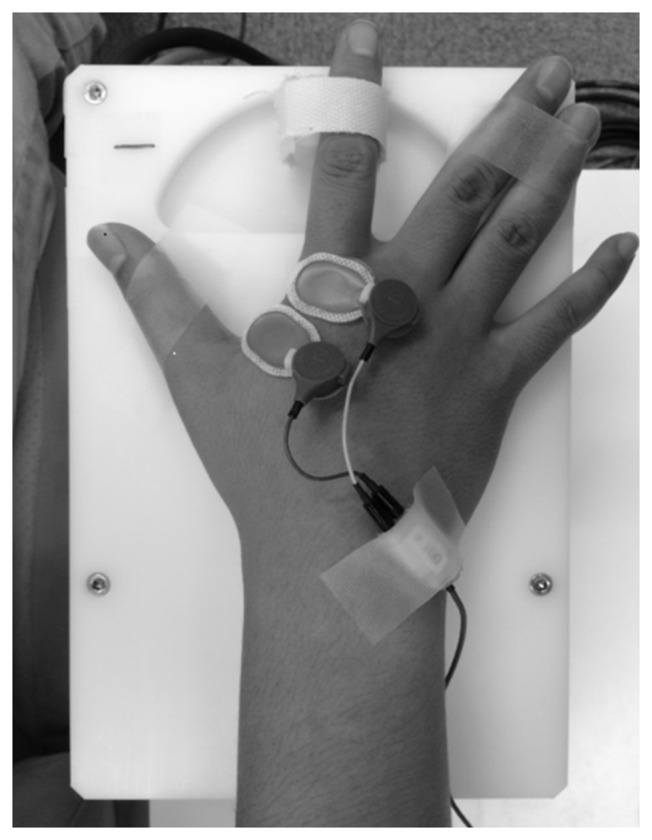
**Passive movement machine**. This machine enables arbitrary setting of range of movement, movement velocity and pause time during passive movement of the index finger. It can also output external triggers at arbitrary joint angles during passive movement.

Passive movement and timing of TMS and electrical stimulation were induced by a dedicated apparatus capable of computer controlled rhythmic movement (Figure 2). Trigger is output at an arbitrary angle set up by the passive movement device. Controlled by a computer, the trigger is output in a random manner during movement at a preset angle. The right index finger was fixed using a strap. The ranges of motion selected for Experiment 1 (10° abduction to 30° adduction) and Experiment 2 (10° adduction to 30° adduction) are below the maximal range of the first metacarpophalangeal joint so that the movements could be performed comfortably. Movement velocity was set at 80°/s, and direction (adduction–abduction) was switched once every 5 s.

### Electromyography (EMG)

Motor responses were recorded from the right FDI using Ag/AgCI electrodes and amplified (A-DL-720-140, 4 Assist, Tokyo, Japan). The recording electrode was attached to the center of the right FDI muscle belly, and the reference electrode was attached to the distal end of the second metacarpal bone. A ground electrode was attached to the right forearm. electromyography (EMG) signals were digitized at 10 kHz, high-pass filtered at 20 Hz, and stored on a personal computer. Waveforms were analyzed using LabChart7 (AD Instruments, Sydney, Australia).

### Transcranial Magnetic Stimulation

MEPs were induced by a Magstimu 200 TMS device (Magstim, Dyfed, UK) with a 95-mm diameter figure-eight coil. The optimal coil position over the left M1 region for each subject, defined as the site eliciting the largest MEP (hot spot), was selecting using TMS Neuro navigation (Eemagine Medical imaging Solutions GmbH, Berlin, Germany) from individual magnetic resonance images. The TMS intensity used was the lowest stimulus intensity that induced an MEP with a peak-to-peak amplitude exceeding 1 mV in the relaxed FDI in at least 5 of 10 consecutive trials, and the stimulation frequency was 0.2 Hz.

### Peripheral Nerve Electrical Stimulation

A peripheral nerve electrical stimulation (SEN-8203, Nihon Kouden, Tokyo, Japan) was used for measurement of M- and F-waves. A stick electrode was used to stimulate the ulnar nerve at the right wrist, and M- and F-waves were measured from right FDI. Stimulation pulses were set to 120% of that inducing maximal M-wave intensity, with 0.2 ms duration and 0.2 Hz stimulation frequency.

### Data Analysis and Statistical Analysis

We confirmed that there was no muscle activity during passive movement by EMG. The mean MEP amplitude was calculated from the peak-to-peak amplitudes of 10 measurements (with the maximum and minimum MEP amplitudes excluded) under each condition. Peak-to-peak M- and F-wave amplitudes were obtained by averaging 50 stimuli under each condition. F-wave persistence was defined as the proportion of F-waves 50 μV or larger. All values are shown as mean ± standard error.

All statistical analyses were conducted using PASW Ver. 21 (SPSS; IBM, Armonk, NY, USA). MEP amplitude, M-wave amplitude, F-wave amplitude and persistence, F/M ratio and MEP/M ratio from Experiment 1 and MEP amplitude from Experiment 2 were compared among conditions (joint angle and passive movement direction) by repeated measures one-way analysis of variance with Dunnett’s tests for pair-wise *post hoc* comparisons. Differences were considered significant at *p* < 0.05 for all analyses. In Experiments 1 and 2, MEP amplitude values during passive movement were normalized based on those obtained at static 0° or 20° of adduction. Wilcoxon signed-rank test was used for comparing static and normalized values. Differences were considered significant at *p* < 0.05 for the analyses.

## Results

**Figure 3:**
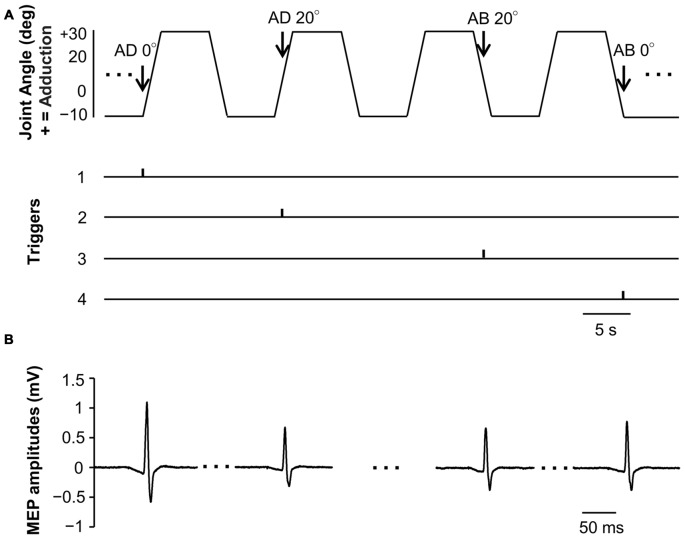
**Joint angle, trigger signals and MEP waveforms elicited by one representative subject during passive movement in Experiment 1. (A)** Abduction-adduction joint angle and output trigger signals for transcranial magnetic stimulation (TMS) during passive movement. Triggers 1, 0° joint angle during passive adduction (AD 0°); Trigger 2, 20° adduction during passive adduction (AD 20°); Trigger 3, 20° adduction during passive abduction (AB 20°); Trigger 4, 0° joint angle during passive abduction (AB 0°). **(B)** MEP waveforms elicited at each joint angle during passive movement.

MEP waveforms of one representative subject recorded during passive movement are as depicted in Figure 3. We used EMG to confirm that there was no activity in the FDI muscle during passive movement of the right index finger.

### Experiment 1–Changes in MEPs, M-waves and F-waves during Passive Movement

**Figure 4:**
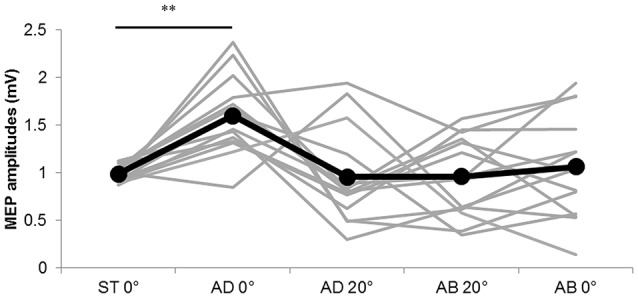
**MEP amplitudes during static and passive movement conditions in Experiment 1**. The black line shows the mean MEP amplitude and gray lines the individual values in Experiment 1 at 0° static joint angle (ST 0°), 0° joint angle during passive adduction (AD 0°), 20° adduction during passive adduction (AD 20°) and the same angles during abduction (AB 0° and AB 20°). There was a significant increase in MEP amplitude at AD 0° compared with ST 0° (**p < 0.01).

The MEP amplitudes measured in Experiment 1 in the neutral position (0° joint angle) at rest (static condition) and at both 0° and 20° adduction during passive adduction (AB 10°–AD 30°) and abduction (AD 30°–AB 10°) are shown in Figure 4. There were significant differences in MEP amplitude and MEP/M ratio during passive movement compared with the static position (MEP: *F*_(4,52)_ = 6.334, *p* = 0.000, partial *η*^2^ = 0.328; MEP/M ratio: *F*_(4,52)_ = 4.515, *p* = 0.003, partial *η*^2^ = 0.258). *Post hoc* testing revealed that MEP amplitude and MEP/M ratio were increased at 0° during passive adduction (AB 10°–AD 30°) compared with the static 0° joint position (*p* < 0.01 for both; Table 1). Alternatively, there were no differences among the other conditions. Similarly, there were no significant differences in M-wave amplitude (*F*_(1.531,19.905)_ = 3.502, *p* = 0.06, partial *η*^2^ = 0.212), F-wave amplitude (*F*_(2.029,26.382)_ = 1.941, *p* = 0.163, partial *η*^2^ = 0.13), F-wave persistence (*F*_(4,52)_ = 0.463, *p* = 0.763, partial *η*^2^ = 0.034), or F/M ratio (*F*_(4,52)_ = 1.632, *p* = 0.18, partial *η*^2^ = 0.112) among static and passive movement conditions (Table 2).

**Table 1:** **Mean motor evoked potential (MEP) amplitude and MEP/M ratio during passive movement in Experiment 1**.

	(*n* = 14)				
	Static	Adduction movement		Abduction movement	
	ST 0°	AD 0°	AD 20°	AB 20°	AB 0°
MEP amplitudes (mV)	0.98 ± 0.02	1.55 ± 0.11**	0.94 ± 0.13	0.93 ± 0.11	1.05 ± 0.14
ME-PIM ratio (%)	10.05 ± 0.75	15.50 ± 0.82**	9.48 ± 1.79	10.53 ± 1.77	10.64 ± 0.84
				mean ± SE **p < 0.01 Dunnett	

**p < 0.01 Comparison between 0° joint angle during passive adduction and static 0°.

**Table 2:** **Mean M-wave amplitude, F-wave amplitude and persistence and F/M ratio during passive movement in Experiment 1**.

	(*n* = 14)				
	Static	Adduction movement		Abduction movement	
	ST 0°	AD 0°	AD 20°	AB 20°	AB 0°
M-wave amplitudes (mV)	11.43 ± 0.96	11.19 ± 0.97	10.73 ± 0.96	10.58 ± 0.94	11.07 ± 0.85
F-wave amplitudes (mV)	0.14 ± 0.01	0.18 ± 0.03	0.14 ± 0.02	0.14 ± 0.02	0.14 ± 0.02
Persistence (%)	47.14 ± 5.04	49.57 ± 7.48	46.00 ± 4.40	44.71 ± 5.43	46.71 ± 6.02
FIM ratio (%)	1.28 ± 0.51	1.58 ± 0.74	1.41 ± 0.76	1.40 ± 0.78	1.34 ± 0.59
				mean ± SE	

### Experiment 2–MEP Amplitude Values during Passive Movement and Static Conditions

**Figure 5:**
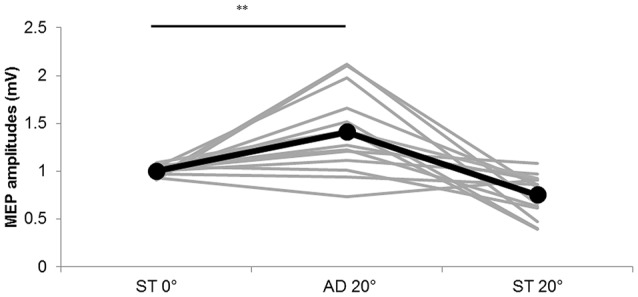
**MEP amplitudes during static and passive movement conditions in Experiment 2**. The black line shows the mean MEP amplitude and the gray lines the individual values in Experiment 2 at 0° static joint angle (ST 0°), 20° adduction during passive adduction (AD 20°) and static 20° adduction angle (ST 20°). MEP amplitude was significantly increased at AD 20° compared with ST 0° (**p < 0.01).

The MEP amplitude values obtained in Experiment 2 are shown in Figure 5. Significant differences among the three conditions were observed (static (ST) 0°: 1.00 ± 0.01 mV, AD 20° during passive adduction: 1.40 ± 0.12 mV, ST 20° adduction: 0.75 ± 0.06 mV; *F*_(1.240,16.124)_ = 16.901, *p* = 0.000, partial *η*^2^ = 0.565), and *post hoc* analysis revealed significantly greater MEP amplitude at AD 20° during passive adduction compared with static (ST) 0° (*p* < 0.01).

### Normalized MEP

As a result of normalization of MEP amplitude values during passive movement based on those obtained at each static angle, the values were 1.63 ± 0.12 (0° during adduction movement), 1.48 ± 0.27 (20° adduction during adduction movement), 1.07 ± 0.14 (0° during abduction movement), and 1.35 ± 0.16 (20° adduction during abduction movement) in Experiment 1. In Experiment 2, the value at 20° adduction during adduction movement was 2.15 ± 0.30. Normalized value significantly increased at only 0° during adduction movement compared with that at 0° static (*p* < 0.01) in Experiment 1. Similarly in Experiment 2, normalized value significantly increased at 20° during adduction movement compared with that at 20° static (*p* < 0.01).

## Discussion

The present study revealed: (1) significantly increased MEP amplitude of the FDI at 0° during passive adduction (10°AB–30°AD) compared with static 0° joint position (Experiment 1) and (2) significantly increased MEP amplitude at 20° adduction during passive adduction (10°AD–30°AD) compared with static 0° (Experiment 2). In contrast; (3) MEP amplitude did not fluctuate during passive abduction; and (4) M-waves and F-waves (both amplitude and persistence) were not influenced by joint angle or passive movement direction. Thus, afferent inputs from passive movement can influence cortical excitability as manifested by MEP amplitudes. However, these effects appeared independent of absolute joint position and passive movement direction. In this case, enhancement may have arisen due to the specific interval between afferent input and TMS, which corresponded to the temporal window of intracortical AF.

In Experiment 1, MEP amplitude increased at 0° during passive adduction compared with static 0°, while there was no significant difference at 20° adduction during passive adduction from 10° abduction. No changes were observed at either 0° or 20° adduction during abduction. However, Experiment 2 revealed that MEPs at 20° adduction were significantly higher than static 0° during passive adduction from 10° adduction. This suggests that the increase in MEP amplitude during passive movement is dependent on the time from start of the movement (start of muscle extension) rather than absolute joint angle or direction of movement. In the present study, 10° passive joint movement required 125 ms as the movement velocity was 80°/s. The MEP facilitation suggests that corticospinal tract excitability had increased by 125 ms from the start of muscle extension. However, no significant difference was detected in the amplitude and persistence of F-waves during passive movement compared with static baseline regardless of joint angle and time from start of movement, so the increase in MEP during passive movement likely occurred at the cortical level instead of the spinal level.

Multiple studies have reported that TMS-induced MEP amplitude is altered by preceding electric stimulation of a peripheral nerve, such as the median nerve, dependent on the ISI (Deletis et al., 1992; Komori et al., 1992; Tokimura et al., 2000; Kotb et al., 2005; Tamburin et al., 2005; Devanne et al., 2009; Degardin et al., 2011). For example, MEPs are attenuated at 20–40 ms ISI, termed SAI or SAI (Tokimura et al., 2000; Kotb et al., 2005; Tamburin et al., 2005; Devanne et al., 2009) but increased at 45–80 ms ISI (AF; Deletis et al., 1992; Komori et al., 1992; Devanne et al., 2009; Degardin et al., 2011). Thus, it is likely that MEP amplitude increased in this study when the delay between passive movement initiation and TMS occurred within the AF temporal window.

However, this 125 ms ISI between the start of passive movement and MEP measurement is longer than the ISIs inducing AF in previous studies using electric stimulation (Deletis et al., 1992; Komori et al., 1992; Devanne et al., 2009; Degardin et al., 2011). It has been reported that the latency of somatosensory evoked potentials is longer when induced by passive movement compared with direct electrical peripheral nerve stimulation (Mima et al., 1996). Thus, passive movement-associated afferent input was likely delayed compared with direct stimulation-induced input, possibly accounting for the longer ISI.

Edwards et al. (2002, 2004), reported that MEP amplitude measured from the FDI decreased during adduction, in direct contradiction to the results of the current study. Though the reason is unclear, Edwards et al. (2002) speculated that the absence of AF in their study may arise from use of a series of repetitive movements. In addition, MEP amplitudes were reported to increase in the direction of muscle contraction during passive movement of the wrist (Lewis et al., 2001), while in our study, no increase in MEP amplitude was observed during abduction of the index finger, which would shorten the FDI muscle. Although the reason for this is unclear, Lewis et al. (2001), Coxon et al. (2005) and Chye et al. (2010) speculated that the reciprocal inhibition pathway between agonist and antagonist muscles of the wrist may be involved. However, we speculate that there may be differences between movement sites (wrist vs. finger) because it was reported that no reciprocal inhibition occurs between agonist and antagonist muscle of the fingers (Hines et al., 1993). From these results, a possibility of involvement at the spinal cord level in case of the wrist joint has been suggested, and it is believed to be difficult to simply compare excitability change at a cortical level.

This study has several limitations. We did not measure the control muscles and could not validate the influence of passive movement that occurred specifically to the main action muscle. In addition, we did not normalize the MEP amplitude at 20° adduction at static. In Experiment 1, MEP did not increase at 20° adduction during adduction movement; in Experiment 2, MEP increased significantly at 20° adduction during adduction movement. Because the value of MEP differs depending on the time from the start of movement even at the same 20° adduction, it is believed that the result of this experiment is unaffected by normalization. Our results suggest that AF rather than absolute joint angle or passive movement direction enhances the MEP amplitude of the FDI. However, we did not test for possible SAI using shorter intervals between movement initiation and MEP induction. Similarly, we did not test short interval intracortical inhibition and intracortical facilitation by paired-pulse TMS (third limitation). Thus, future studies are required to evaluate cortical facilitatory and inhibitory circuits.

In summary, we demonstrate corticospinal excitability is not influenced by either joint angle or direction of movement. Rather, the MEP increases observed 125 ms after the start of muscle extension likely reflect intracortical AF induced by passive movement-associated afferent input.

## Author Contributions

HO and MN conceived the study and designed the experiments. MN and RS conducted the experiments. SM and SK performed interpretation of data. MN and ST performed the statistical analysis. KS and YI helped writing the manuscript. HO and MN wrote the manuscript. All authors read and approved the final manuscript.

## Conflict of Interest Statement

The authors declare that the research was conducted in the absence of any commercial or financial relationships that could be construed as a potential conflict of interest.
